# About Bills in the Hands of a Collection Agency

**Published:** 1894-12

**Authors:** 


### ABOUT BILLS IN THE HANDS OF A COLLECTION AGENCY.

Last year we stamped on each subscriber's bill, where the amount was ten dollars or more, a special offer of settlement. Not one responded to the offer.

We have always been willing, and so published, to accept any correction of his account which a subscriber made; and have made all corrections asked by subscribers. We are yet willing to do so, and protect the subscriber.

The Publishers' Collection Agency has been given a list of all subscribers whose account appeared on the books as $12.00 or more, because each one of them had the chance last year to settle on his own terms, and not only did not attempt but continued to receive THE JOURNAL.

If the account is wrong we shall gladly correct it. If misfortune has prevented a settlement, we shall protect you as far as possible. Where the account has simply been neglected, the matter is beyond our reach and you will have to settle with the agency.

BUSINESS MANAGER.

---

THE Seventh Annual meeting of the Southern Surgical and Gynecological Association was held in the German Artillery Hall, Charleston, S. C., November 13th, 14th, and 15th. The attendance, while not large, represented some of the strongest men in the profession. Members were present from New York, Philadelphia, Baltimore, Washington, Richmond, Columbia, Atlanta, Chattanooga, Nashville, Louisville, New Orleans, and many smaller cities and towns.

A number of excellent papers were read and the discussions were unusually good. The Charleston profession entertained the Association in a royal manner. An elegant lunch was served each day in the hall. The second afternoon was spent in a boat-excursion around the harbor, where many historic places were seen.

The officers for next year are: President, Louis McLane Tiffany, Baltimore; First Vice-President, E. S. Lewis, New Orleans, La.; Second Vice-President, Maning Simons, Charleston; Secretary, W. E. B. Davis, Birmingham, Ala.; Treasurer, Richard Douglass, Nashville, Tenn.

The next meeting of the Association will be held in Washington, D. C., on the second Tuesday in November, 1895.

