# An Error

**Published:** 1895-01

**Authors:** 


AN ERROR.—In December issue, sixth line from top of page 624, "did not *attempt*" should have been printed "did not *accept*."

